# Planning for work: Exploring the relationship between contraceptive use and women’s sector-specific employment in India

**DOI:** 10.1371/journal.pone.0248391

**Published:** 2021-03-11

**Authors:** Lotus McDougal, Abhishek Singh, Kaushalendra Kumar, Nabamallika Dehingia, Aluisio J. D. Barros, Fernanda Ewerling, Yamini Atmavilas, Anita Raj

**Affiliations:** 1 Center on Gender Equity and Health, School of Medicine, University of California San Diego, La Jolla, California, United States of America; 2 International Institute for Population Sciences, Mumbai, Maharashtra, India; 3 International Center for Equity in Health, Universidade Federal de Pelotas, Pelotas, Rio Grande do Sul, Brazil; 4 Bill and Melinda Gates Foundation, New Delhi, Delhi, India; University of Botswana, BOTSWANA

## Abstract

While the health-related benefits of contraceptive use for women are well documented, potential social benefits, including enabling women’s employment, have not been well researched. We examine the relationship between contraceptive use and women’s employment in India, a country where both factors have remained relatively static over the past ten years. We use data from India’s 2015–16 National Family Health Survey to test the association between current contraceptive use (none, sterilization, IUD, condom, pill, rhythm method or withdrawal) and current employment status (none, professional, clerical or sales, agricultural, services or production) with multivariable, multinomial regression; variable selection was guided by a directed acyclic graph. More than three-quarters of women in this sample were currently using contraception; sterilization was most common. Women who were sterilized or chose traditional contraception, relative to those not using contraception, were more likely to be employed in the agricultural and production sectors, versus not being employed (sterilization adjusted relative risk ratio [aRRR] = 1.5, p<0.001 for both agricultural and production sectors; rhythm aRRR = 1.5, p = 0.01 for agriculture; withdrawal aRRR = 1.5, p = 0.02 for production). In contrast, women with IUDs, compared to those who not using contraception, were more likely to be employed in the professional sector versus not being employed (aRRR = 1.9, p = 0.01). The associations between current contraceptive use and employment were heterogeneous across methods and sectors, though in no case was contraceptive use significantly associated with lower relative probabilities of employment. Policies designed to support women’s access to contraception should consider the sector-specific employment of the populations they target.

## Introduction

Contraceptive choice is a critical component of women’s reproductive health and rights [1]. The health benefits of contraceptive use for women and their children are well documented, and include reduction of unintended pregnancies, pregnancy-related morbidity and mortality, delayed age at first birth among young women, and lengthened birth intervals [1–9]. In contrast, the social and economic correlates of contraceptive use are less well understood, particularly in low- and middle-income countries, where contraceptive use is often assessed in the context of maternal and child health interventions [6].

Women’s ability to control their own fertility has been identified as an important enabler of women’s labor force participation in some settings [10–14]. This relationship is of growing interest, due in no small part to the Sustainable Development Goals [15]. While much of the research exploring this connection has focused on contraceptive use as a consequence of women’s labor market participation [16–19], there are a number of plausible mechanisms suggesting that the reverse may also hold true. At the societal level, as nations experience the demographic transition (in which both birth and mortality rates decline), the resultant dividend offers greater opportunities (and financial benefits) for economic engagement [20,21]. At the individual level, there are data from multiple settings suggesting that childbearing generally depresses women’s labor force participation [10–12,22–24], due to both reduced opportunities for education (in the case of early childbearing) [22,25,26], and the time demands of child care and rearing [27,28]. From a rights-based perspective, access to contraceptives can empower women by reducing uncertainty about timing of pregnancies, thus giving them more control over their own bodies and empowering them to make strategic decisions about their labor force participation [10,29,30].

Despite these theoretical underpinnings, our understanding of the associations between contraceptive use and economic engagement is limited in terms of both nuance and geography. Existing research exploring this relationship has generally conceptualized contraceptive use and/or women’s economic engagement in the aggregate [10–13], when both measures are in fact highly diverse in many populations. Contraceptive use and method choice are influenced by a wide variety of factors, from reproductive history, to sociodemographic characteristics and gender equity such as women’s decision-making agency and power within a relationship [31–34]. Employment is highly heterogeneous in terms of occupation, working conditions, benefits and wages, as well as the extent of women’s choice and agency in whether they work and in what sort of work they engage [35,36]. This suggests that construing women’s economic engagement as a binary construct (e.g. working vs. not working), or even slightly more nuanced categorizations (e.g. formal vs. informal vs. unpaid work) may be inadequate ways of conceptualizing what in practice are unique and diverse experiences [37,38].

Understanding of the relationship between contraception and employment at these more granular levels in India is very limited. India is an important context in which to examine this issue, owing to a unique, sterilization-skewed contraceptive method mix, low and stagnant female labor force participation and widespread gender inequalities [30,39–43]. Contraceptive use among married women aged 15–49 in India declined by 7 percentage points between 2005–06 and 2015–16 (from 64% to 57%), with female sterilization comprising more than 60% of current use, and long-acting reversible contraceptives (e.g. intrauterine devices, injectables) comprising 4% of current use [39].

Similarly, past year employment among married Indian women aged 15–49 declined by 12 percentage points (from 43% to 31%) between 2005–06 and 2015–16 [39,44]. Time poverty amongst working women in India is particularly acute [45,46]. As a result of strong gender asymmetry in the division of domestic labor, most working women in India still spend a substantial amount of their time on housework and caring for their children [45,47–49]. Many women opt to withdraw from the labor force entirely in order to take care of their children, or alternately, choose an occupation that is more accommodating of their prescribed gender roles [50]. Additionally, the Indian labor force is diversified, particularly for non-agricultural employment; there are distinct levels of job availability and demand, wage gaps, working conditions and necessary qualifications across different sectors [51,52]. There are thus diverging plausible pathways for the relationship between contraceptive use and employment in India, further supporting the need for a more detailed analysis of this association.

This study adds to the growing body of research exploring whether and how supporting reproductive health may enable women’s economic engagement [13,53]. We aim to fill gaps in the understanding of the relationship between contraceptive use and women’s employment by analyzing this association in the distinct and important context of India, and by employing more granular definitions of contraceptive use and women’s employment. Our specific objective is to test the association between method-specific contraceptive use and sector-specific employment among married women in India, to identify whether there are variations in this relationship based on type of contraceptive used and sector of employment.

## Methods

Data were drawn from the 2015–16 National Family Health Survey (NFHS-4), a nationally-representative, household-based survey assessing a broad range of sociodemographic, reproductive and health factors [39]. In total, 699,686 women between the ages of 15–49 were interviewed as part of NFHS-4 (response rate of 97%). In 15% of households, women received additional questions including a module on women’s employment. The final analytic sample for this study was restricted to women aged 18–49 years who responded to these employment questions, were currently married and potential contraceptive users (neither infecund [54] nor pregnant at the time of interview, and had not given birth in the 12 months prior to interview), and had no missing values for assessed variables (n = 48,232). Women aged 15–17 were excluded to allow for completion of the window of risk for early marriage (marriage <18 years of age).

### Measures

#### Dependent variable

The main outcome of interest was women’s current paid employment by sector (no current work vs. sector-specific employment. Current employment was defined by answering yes to any of the following questions: “Aside from your own housework, have you done any work in the last seven days?”, “As you know, some women take up jobs for which they are paid in cash or kind. Others sell things, have a small business to work on the family farm or in the family business. In the last seven days, have you done any of these things, or any other work?”. Women who responded yes to either of these questions were then asked whether they were paid and what their occupation was. This analysis focuses on employment, defined as work done for others for payment, in line with international definitions [55]; women were considered to have been paid if they indicated that their earnings were either cash or cash and in-kind. Unemployment could not be defined for this population, as employment-seeking activities were not captured in the data [55].

Occupations were assessed by the question “What is your occupation, that is, what kind of work do you mainly do?”. These occupations were then grouped into five employment sectors: professional (including professional, technical, administrative and managerial occupations); clerical or sales; agricultural; services; and production (including skilled and unskilled manual occupations) (please see S1 Table for more details). These groupings followed classification in the Indian National Classification of Occupations and were provided with the data [39,56], with the exception of clerical or sales workers, which were originally separate groups that were aggregated for this analysis due to similar occupations and low sample sizes. Occupations within each sector were also labelled and provided with the dataset, and are summarized in S1 Table and as follows. The most common occupations in the professional sector were teacher (51%) and nursing and medical and health technicians (17%). The most common occupations in the clerical or sales sectors were saleswomen, shop assistants and related workers (30%), merchants and shopkeepers (20%) and sales workers (17%). In the agriculture sector, most workers were agricultural laborers (74%). In the services sector, the most common occupations were service workers (28%), cooks, waiters, bartenders and related workers (18%) and housekeepers, matrons and stewards (16%). In the production sector, the most common occupations were laborers (40%) and tailors, dress makers, sewers, upholsterers and related workers (30%).

#### Independent variable

The independent variable of interest was current contraceptive use, which was assessed by method: none, female sterilization, condom, pill, IUD, rhythm method and withdrawal. Use of male sterilization, injections, lactational amenorrhea, female condom and foam were excluded based on low prevalence (each <0.5%).

#### Covariates

There are a number of reproductive, sociodemographic and gender inequality factors that plausibly influence the relationship between contraceptive use and employment in India [57,58]. For reproductive history, covariates included parity (0,1,2,3 or more births), and whether the respondent had any living sons (none, any). India has a long history of son preference [59,60], and there is evidence not only of sex-selective abortion, but of avoiding contraception until a son is born. At that point, women may gain both increased status and greater autonomy to leave the house, facilitating opportunities to engage in paid employment [12,13,30,42].

Sociodemographic factors, such as household wealth, have also been shown to influence contraceptive use and employment in numerous settings, including India [12,13,61–63]. Sociodemographic covariates included the woman’s age (in years); woman’s education (in years); number of household residents; household dependency percentage; geographic region; area (urban vs. rural) of residence; scheduled caste (SC), scheduled tribe (ST) or other backwards classes (OBC) (SC/ST, OBC or neither) and household wealth quintile (poorest, poorer, middle, richer, richest). Scheduled caste, scheduled tribe and other backwards class are constitutionally defined groups indicative of pervasive disadvantage and marginalization, have different contraceptive use patterns than other populations in India, and are targets of employment quotas [39,64]. Household wealth quintile was provided with the dataset, and is an index based on household assets and characteristics. It is estimated through principal components analysis and adjusted for the different assets available in urban and rural areas [65]. Household dependency is generally expressed as a ratio of the number of dependents in the household (children under 15 years of age and adults aged 65 or older) to the number of people of working age (15–64) in the household [66]. To facilitate interpretation, we have instead used household dependency percentage, calculated as the number of dependent individuals in the household (under 15 or over 64 years of age) divided by the total number of individuals in the household. Higher percentages therefore indicate higher dependency, and vice versa.

Gender equity, including spousal power dynamics, may be influenced by both women being married at young ages, and by differential levels of education, thus affecting household bargaining power, the ability to make decisions about contraception and the desire, need and ability to work [37,40,67,68]. Gender equity covariates include age at first marriage or cohabitation (under 15, 15–17 and 18 or older); marital education gap (wife more educated, equally educated, husband more educated); freedom of movement, based on whether the respondent was able to go alone to the market, to a health facility and to places outside her village or community, or if she could not go/required accompaniment to one or more of these locations (restricted access/accompaniment to at least one of three locations, freedom of movement to all three locations); and decision-making involvement, that measured whether the respondent was involved in decision making (either solely or with husband/partner) about health care for herself, major household purchases and visits to family or relatives (not involved in at least one decision, involved in all three decisions).

### Analysis

**Fig 1:**
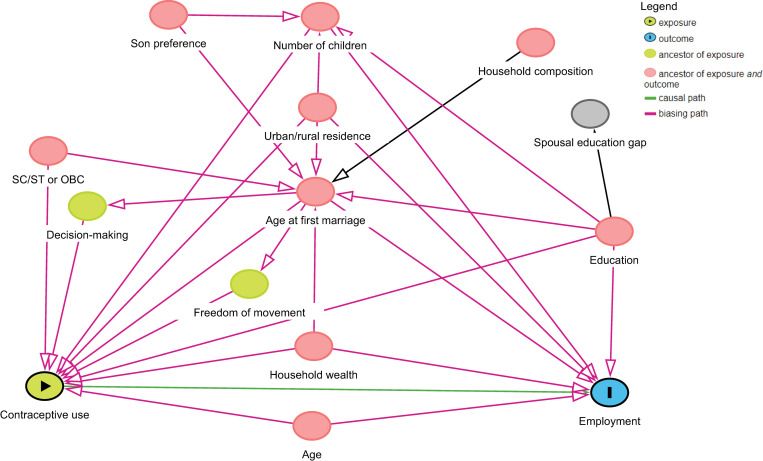
Direct acyclic graph model of causal assumptions used for covariate selection in modeling the relationship between contraceptive use and employment in India. Notes: Scheduled caste, scheduled tribe and other backwards class are constitutionally defined groups indicative of pervasive disadvantage and marginalization. Household composition is a measure of household dependency, assessing the percentage of dependent (very young and very old) individuals as a percentage of all household residents.

Descriptive analyses explored the distribution of current contraception and covariates by employment sector. Recognizing the large number of plausible confounders identified, we created a directed acyclic graph (DAG) model to reduce bias, improve precision and transparency, and guide multivariable regression variable selection (Fig 1).

DAGs are visual tools used to represent hypothesized, causal relationships (depicted by unidirectional arrows) between variables (depicted by nodes). The DAG pathways in Fig 1 were created based on existing research, as well as authors’ topical and theoretical expertise [69–71]. Reviewed research covered all assessed variables, including parity [40,72], son preference [34,72], age [11,31,40], urban/rural residence [12,73], education [12,37,72,74], SC/ST or OBC status [40,72,75], household wealth [31,72,76], household composition [77], age at first marriage [32,33,67,74,78,79], spousal education gap [12,80], freedom of movement [72,78], and decision-making [78,81].

Pathways represented along DAGs can be analyzed to identify the smallest subset(s) of variables possible that must be included in the model in order to sufficiently adjust for confounding (generally termed a minimal sufficient adjustment set) [69,70,82]. Following methods described by Shrier and Platt [69,82], the minimal sufficient adjustment set of variables for the DAG presented in Fig 1 comprised age, age at first marriage, education, household wealth, number of children, and area of residence. These variables were then included in multivariable, multinomial logistic regression models testing the associations between current contraceptive use and current employment status/sector. Multicollinearity among independent variables was not present based on a VIF cutoff of >4. Multivariate models also included state/union territory fixed effects, to account for state-specific variation not addressed by covariates, as well as a squared term for age to account for the non-linear relationship between age and employment. Multinomial logistic models provide relative risk ratios as coefficients, which are ratios of the odds (or relative risk) of a one-unit change that a given covariate has on a given outcome as compared to that probability for the base (reference) outcome.

Analyses were conducted using Stata (StataCorp. 2019. *Stata Statistical Software*: *Release 16*. College Station, TX: StataCorp LLC) and R (version 3.6.1). DAGitty 3.0 was used to create the DAG [82]. All analyses were adjusted for survey design and sample weights. Ethical approval for survey design, questionnaires and data collection was obtained by the International Institute for Population Sciences and the ICF Institutional Review Board. Ethical approval for this analysis was provided by the University of California San Diego Institutional Review Board.

## Results

### Descriptive results

**Fig 2:**
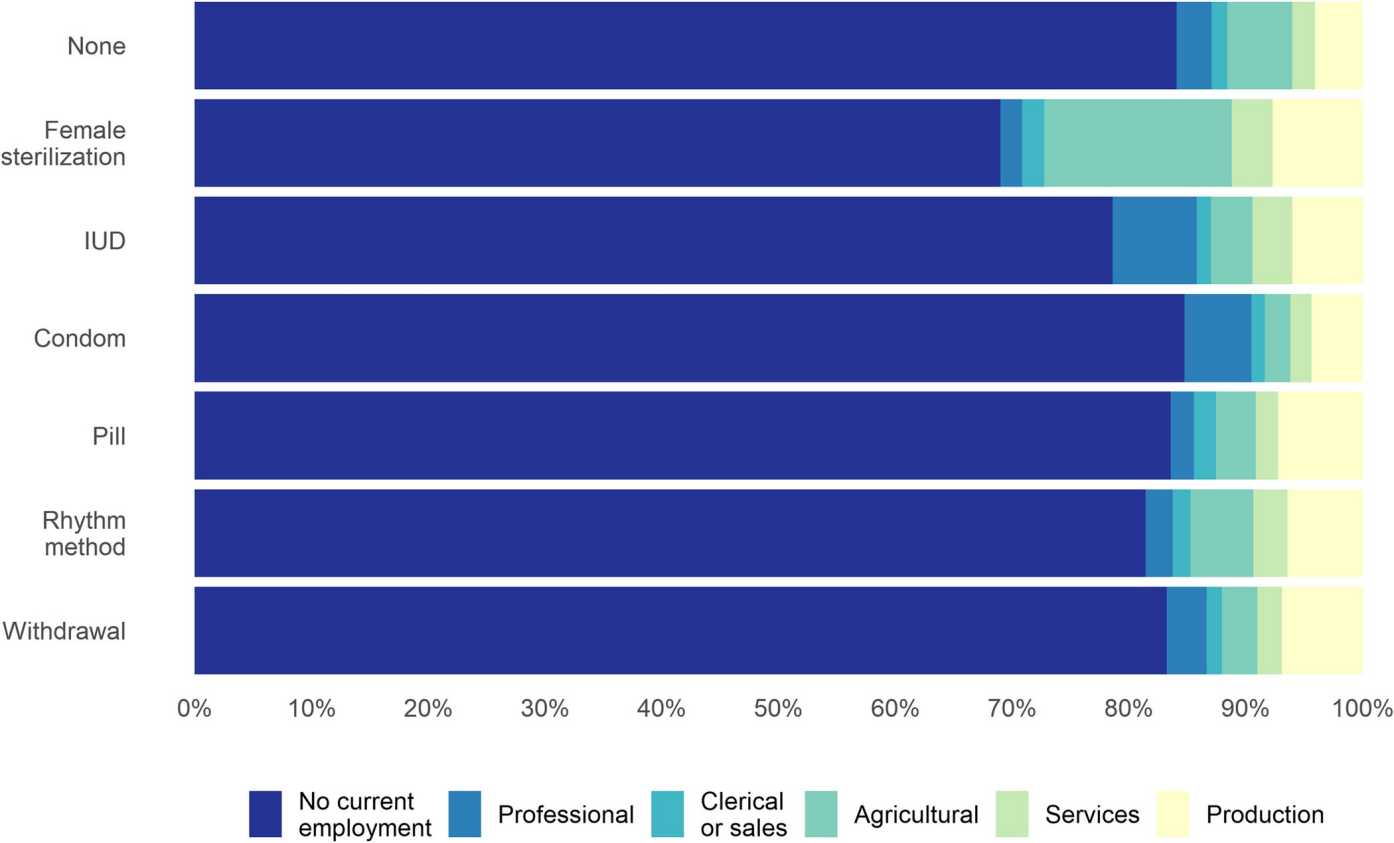
Descriptive summary of current contraceptive use across current employment sectors among women in India.

Nearly one in four women (24%) were not currently using any contraception (S2 Table). Among these non-users, 84% were not currently employed, and 6% worked in the agricultural sector (Fig 2 and S2 Table). There was substantial variation in contraceptive methods, though female sterilization was the most common form of contraception used (52% of respondents). Among women who had been sterilized, the majority (69%) were not currently employed, 16% were employed in the agricultural sector, and 8% were employed in the production sector. Among the 8% of the sample who reported current condom use, 85% were not currently employed, 6% were employed in the professional sector, and 4% were employed in the production sector. Amon the 2% of the sample who were current IUD users, 79% were not currently employed, 7% were employed in the professional sector, and 6% were employed in the production sector.

One in four analyzed women (24%) were employed at the time of interview (S2 Table). In comparison with currently employed women, women who were not employed tended to be younger, have more people living in their households, have husbands who were more educated than themselves, and to have more restricted freedom of movement and less involvement in decision-making. The prevalence of being not currently employed was highest among women who had had no births and who were in the richest household wealth quintile; this prevalence decreased as women had more children, and as household wealth decreased. The prevalence of having two or more births was much higher for women employed in the agricultural and production sectors, and was lower for women in the professional sector. The prevalence of working in the professional sector increased as household wealth increased, while the prevalence of working in the agricultural sector decreased as household wealth increased. Education levels were lowest among agricultural and production sector workers (an average of 2.9 and 4.9 years of education, respectively), and highest among professional and clerical or sales sector workers (13.7 and 7.8 years, respectively).

### Regression results

**Fig 3:**
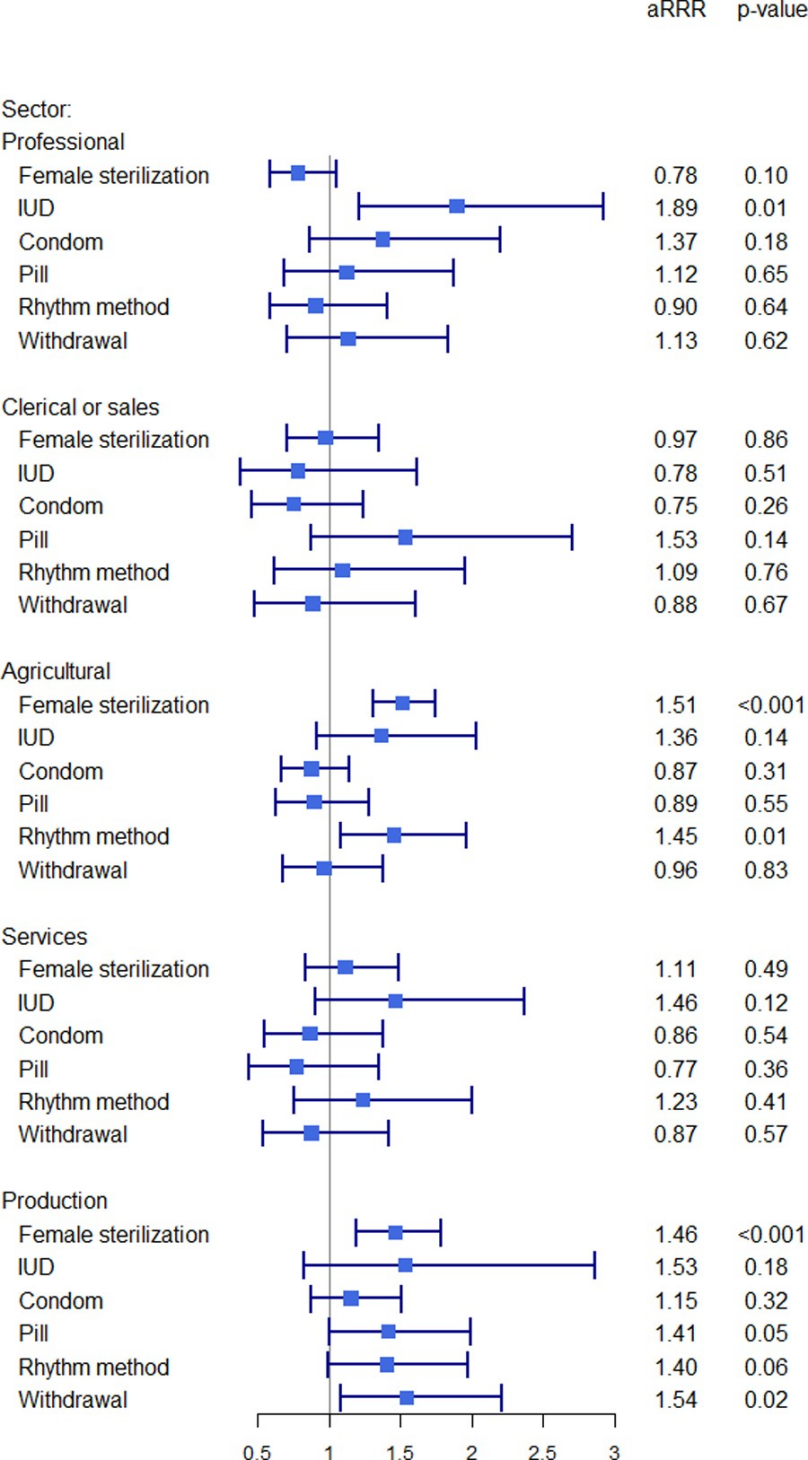
Multinomial associations between type of current contraception and current employment among women in India. Notes: Outcome reference group is no current employment; reference group for contraception is no current contraception. aRRR = adjusted relative risk ratio. Model adjusts for parity, age, education, residence, household wealth, and age at first marriage/cohabitation, as well as age squared and a state/union territory fixed effect.

Multivariable, multinomial regression analyses show that for IUD users, relative to women not currently using contraception, the relative risk of current employment in the professional sector (vs. not being employed) was 1.9 times greater (p = 0.01) (Fig 3 and S3 Table). Women who were sterilized were more likely to be employed in the agricultural and production sectors (aRRR = 1.5, p<0.001 for each) as compared to not being employed. Women currently using the rhythm method were likely to be employed in the agricultural sector (aRRR = 1.5, p = 0.01). Finally, women currently using withdrawal were more likely to be employed in the production sector (aRRR = 1.5, p = 0.02). In no case was contraceptive use significantly associated with lower likelihood of employment.

Parity was generally not associated with current employment, excepting that relative to nulliparous women, women with children were less likely to be employed in the professional sector (S3 Table). With regards to sociodemographics, age was consistently and positively associated with employment, irrespective of sector. Education was positively associated with employment in the professional, clerical or sales, and services sectors, and negatively associated with employment in the agricultural and production sectors. Rural residents had a much higher likelihood of agricultural employment, and urban residents had a higher likelihood of clerical or sales, services, and production employment. Generally, as levels of household wealth decreased, the likelihood of employment in all sectors barring clerical or sales increased.

In terms of gender equity, women who married very young (<15) had higher probabilities of being employed in all sectors save professional, compared to those who married at age 18 or later (S3 Table).

## Discussion

More than three-quarters of women in this sample were current contraceptive users. While that use was dominated by female sterilization, there was great variability across methods and employment sectors. Generally, contraceptive use was positively associated with employment; in no assessed group was contraceptive use significantly associated with lower relative probabilities of employment. There was, however, substantial variation in the relationship between contraceptive use and employment, based on the type of contraceptive used, and sector of employment. This heterogeneity underscores the importance of looking at contraception and employment as disaggregated variables where possible, to avoid masking important and distinct associations.

Several profiles of contraceptive use across different employment sectors emerged from this analysis, with important programmatic implications. Though women in the sample had similar mean ages across sectors, and employment–irrespective of sector—increased with age, women using the only long-acting reversible contraceptive reported (IUD), and women with no children, were more likely to be employed in the professional sector than to not currently be employed. In contrast, women reporting female sterilization or non-modern methods (e.g. rhythm, withdrawal) were more likely to be employed in the agriculture or production sectors than to not currently be employed. Women employed in the agricultural and production sectors also tended to have more children than other employed women in this sample, though this was not a significant predictor in multivariable modeling.

These distinctions fall in part along the division of formal and informal employment. Women in the professional sector were primarily teachers and nurses, largely a part of the formal economy. The informal economy, which comprises nearly 90% of Indian employment, is heavily represented in both the agricultural sector (primarily agricultural laborers in this sample) and the production sector (primarily [non-agricultural] labor, textile and garment work, and tobacco work in this sample) [83,84]. While many occupations in the clerical or sales, or services, sectors are also encompassed within the informal economy, the occupations noted by women working in these sectors in our sample focus more on the provision of services than target-based production and labor [84,85]. There was no association between contraceptive use and employment in either the clerical or sales, or services, sector in our analyses.

The patterns of contraceptive use in these formal vs. informal sectors of employment suggest that, for these two groups of women, there may divergent pathways for contraceptive use with regards to employment: control over childbearing, in which women have fewer children and use highly effective, long-acting reversible contraception, versus methods not requiring regular health system interactions, in which women opt for permanent cessation of childbearing or use of less effective, traditional methods [86]. The fact that sterilization and traditional methods were significantly associated with employment in agricultural and production sectors even after adjusting for area of residence and wealth suggests that supply-side access may not be a barrier, but that demand and preference for these methods in this population exists, as has been documented elsewhere [87]. This merits further research, particularly to understand why women working in agricultural and production sectors opt for traditional methods, in addition to sterilization.

The divergence between contraceptive use in the professional sector vs. in the agricultural and production sectors may also be indicative of differential family leave policies and flexible work arrangements. India has had paid maternity leave since 1961, but that leave is applicable only to businesses with 10 or more employees, thus lacking coverage for informal laborers and small business employees [88]. This coverage has recently been expanded to offer a longer leave duration, an option to work from home, and childcare facilities in larger establishments, but women in the informal economy remain without benefits [89]. Use of permanent contraception in particular may thus respond to the lack of parent-friendly work environments by facilitating employment in largely informal sectors with high production targets, long work hours, low wages, lack of parental leave, and verbal abuse [90,91]. This is perhaps particularly unsurprising in a context where women bear 90% of the burden of unpaid care work [92].

Women with more education were more likely to be employed in all sectors than to not be employed, with two key exceptions: lower levels of education were associated with employment in both agricultural and production sectors. This confluence of lower levels of education paired with higher levels of sterilization and less effective traditional contraceptive methods predicting employment in agricultural and production work again emphasizes a vicious cycle of employment and opportunity; women may not have the education necessary to seek employment in jobs offering greater social protections in terms of childbearing and childrearing, as well higher wages with a less stark wag gap, as well as facing a paucity of time and resources with which to seek that additional education and professional training [13,14,84,90,93].

Compared with women in the most affluent household wealth quintile, poorer women were generally more likely to be employed in any sector than to not be currently employed, with the largest relative risk ratios in the agricultural sector. The exception was women working in the clerical or sales sector, where there was no association between household wealth and employment. The fact that this inverse relationship holds true across four of five sectors is a direct confirmation that in Indian society, it is generally poorer women who work, indicating that women’s employment in India may more often be a measure of need and poverty rather than a productive expression of professional interest. This may be compounded by the wage gap between men and women, which tends to be exacerbated in unskilled jobs [51,93].

This relatively linear relationship between wealth and employment is not in line with the U-shaped curve that is often seen with women’s labor force participation, in which lower levels of economic development are associated with higher levels of women’s employment based on financial need, moderate levels of economic development are associated with lowered female labor force participation as financial need at the individual and household levels decreases, and higher levels of economic development as associated with increased female labor force participation commensurate with increased education levels and incomes (as well as the ability to pay for child care when needed) [37,40,94]. India is somewhat of an outlier in this relationship, with many possible explanations for the current stagnating levels of female employment, including subnational heterogeneity, rising household incomes, inadequate job opportunities and social norms that do not support women working [37,40]. While women’s labor force participation is influenced by multiple factors on the supply and demand side [43], a comprehensive review is outside the scope of this paper.

Women who were married prior to age 18, a known health risk and human rights violation [67], had higher probabilities of being employed in all sectors save professional. Women and girls who are married early are more likely to experience lower levels of power within their relationships, and to be poorer [67]. Given that post-marital education is quite rare in this context, the agency to decide whether to work, and the opportunity to gain relevant job skills for employment in the professional sector may thus be compromised for this group of women [74].

This analysis has important limitations. While the use of a DAG, inclusive of causal assumptions, is helpful in reducing bias, these data are cross-sectional in nature, and causality cannot be presumed [71]. The rationale for whether to use contraception, and if so what method, as well as further information on why women did or did not work (and in what sector) are not assessed in these data, and merit further research. This analysis examined only work that was done for pay. Six percent of interviewed women worked but were not paid in cash, and were thus excluded from this analysis. These unpaid women worked primarily in agriculture, and were significantly different from paid women across nearly all covariates. While this unpaid cadre of working women is of great importance, their socioeconomic, demographic and equity differences from paid, working women render them outside the scope of this paper. Employment status was assessed at the time of interview, and there is no way of ascertaining the duration of employment in a particular job or sector from these data. Finally, all data are self-reported, and thus subject to recall and social desirability bias.

One of many benefits of contraception is that women’s control over their own reproduction may enable participation in the labor market [10,13,14]. However, in India, contraception was historically used for reproductive completion, rather than reproductive control [95,96]. In line with the general population in India, contraceptive use in this sample was dominated by female sterilization. Changing this deep-seeded cultural norm is an ongoing process, and there are clearly populations that require additional attention and support. Despite government efforts to increase IUD utilization, uptake decreased from 3.2% to 2.3% over the last decade, and the prevalence among women in this sample was only 2.1% [39,97–99]. It is clear that efforts to expand the method mix to include long-acting, reversible contraceptives are leaving important populations of women behind, and need additional targeted support. The variable associations between types of current contraceptive use and employment by sector seen in this analysis emphasize what a complex interplay is at work here. The most stark contrast, between women employed in professional vs. agricultural and production sectors, suggests that even after accounting for differential levels of social and gender equity, women who were sterilized or relied on traditional contraceptives were more likely to be employed in the agricultural or production sectors, and women using long-acting, reversible contraception, which overall has low prevalence of usage in India, were more likely to be employed in the professional sector. Both qualitative and longitudinal research are needed to further explore these dynamics.

## Conclusions

Women’s labor force participation in India has been generally declining for more than a decade [83]. Contraceptive access can be one means by which to support women’s employment [10], but recognition is needed that a one-size-fits-all approach is unlikely to be successful at enabling women to make safe choices about if, when and how many children to have. It is also critical not to conflate women’s economic engagement with women’s economic empowerment–the latter implies choice, opportunity and decision-making that are possible, but not necessitated, in the former. A more nuanced understanding of whether family planning interventions should be considered a potential enabler for economic engagement will inform multisectoral approaches to health and development. Policies targeting both increased contraceptive utilization and labor force participation will need to consider not only which types of contraception a woman is considering using and what her employment status is, but also the broader social and gender equity context in which these decisions are being made.

## Supporting information

S1 Table Occupations by employment sector among married women aged 18–49 in India, 2015–16. (DOCX)

S2 Table Descriptive summary of variables across current employment sector among 18–49 year old women in India, 2015–16. (DOCX)

S3 Table Multivariable, multinomial regression assessing the associations between current contraceptive use and current employment sector among married women aged 18–49 in India, 2015–16. (DOCX)
