# Supplementary material for: Planning for work: Exploring the relationship between contraceptive use and women’s sector-specific employment in India
Source: PLoS One. 2021 Mar 11;16(3):e0248391. doi: 10.1371/journal.pone.0248391 (PMC7951869; doi:10.1371/journal.pone.0248391)
Supplement: S1 Table — (DOCX) [file pone.0248391.s001.docx]

**S1 Table. Occupations by employment sector among married women aged 18-49 in India, 2015-16.**

|  | Occupations | Prevalence (within sector) |
| --- | --- | --- |
| **Professional (n=1,232)** | | |
|  | Teachers | 51.2% |
|  | Nursing and other medical and health technicians | 17.4% |
|  | Accountants, auditors and related workers | 4.1% |
|  | Professional workers, n.e.c.^1^ | 2.9% |
|  | Physical scientists | 2.8% |
|  | Sculptors, painters, photographers, and related creative artists | 2.8% |
|  | Social scientists and related workers | 2.7% |
|  | Architects, engineers, technologists and surveyors | 2.5% |
|  | Scientific, medical and technical persons, other | 1.8% |
|  | Administrative and executive officials government and local bodies | 1.5% |
|  | Jurists | 1.4% |
|  | Administrative, executive and managerial workers, n.e.c. ^1^ | 1.1% |
|  | Life science technicians | 1.0% |
|  | Aircraft and ships officers | 1.0% |
|  | Engineering technicians | 0.8% |
|  | Economists, and related workers | 0.8% |
|  | Physicians and surgeons | 0.8% |
|  | Physical science | 0.6% |
|  | Mathematicians, statisticians and related workers | 0.6% |
|  | Elected and legislative officials | 0.5% |
|  | Poets, authors, journalists and related workers | 0.5% |
|  | Life scientists | 0.4% |
|  | Working proprietors, directors and managers, wholesale and retail trade | 0.3% |
|  | Directors and managers, financial institutions | 0.3% |
|  | Working proprietors, directors and managers mining | 0.2% |
|  | Working proprietors, directors managers and related | 0.2% |
|  | Working proprietors, directors and managers, other services | 0.1% |
|  | Composers and performing artists | 0.1% |
| **Clerical or sales (n=770)** | | |
|  | Salesmen, shop assistants and related workers | 29.7% |
|  | Merchants and shopkeepers, wholesale and retail trade | 20.1% |
|  | Sales workers, n.e.c. ^1^ | 16.8% |
|  | Clerical and other supervisors | 7.5% |
|  | Village officials | 4.3% |
|  | Insurance, real estate, securities and business service, | 3.9% |
|  | Manufacturers, agents | 3.6% |
|  | Clerical and related workers | 3.6% |
|  | Technical salesmen and commercial travelers | 3.4% |
|  | Computing machine operators | 2.6% |
|  | Money lenders and pawn brokers | 1.3% |
|  | Book keepers, cashiers and related workers | 1.1% |
|  | Stenographers, typist and card and tape punching operators | 0.7% |
|  | Transport and communication supervisors | 0.4% |
|  | Mail distributors and related workers | 0.4% |
|  | Transport conductors and guards | 0.3% |
|  | Telephone and telegraph operators | 0.3% |
| **Agriculture (n=4,641)** | | |
|  | Agricultural laborer | 74.4% |
|  | Farmers, other than cultivators | 12.7% |
|  | Cultivators | 5.5% |
|  | Other farm workers | 2.9% |
|  | Plantation laborers & related workers | 2.3% |
|  | Forestry workers | 0.8% |
|  | Farm plantation, dairy and other managers and supervisors | 0.7% |
|  | Fishermen and related workers | 0.5% |
|  | Hunters and related workers | 0.1% |
| **Services (n=1,228)** | | |
|  | Service workers | 27.8% |
|  | Cooks, waiters, bartenders and related workers (domestic & institutional) | 18.2% |
|  | Housekeepers, matrons and stewards (domestic & institutional) | 16.1% |
|  | Maids and related housekeeping service workers, n.e.c. ^1^ | 10.1% |
|  | Building caretakers, sweepers, cleaners and related workers | 7.2% |
|  | Protective service workers | 7.0% |
|  | Hair dresser, barbers, beauticians and related workers | 5.7% |
|  | Launderers, dry-cleaners and pressers, N.E.C. ^1^ | 4.1% |
|  | Hotel and restaurant keepers | 3.7% |
| **Production (n=2,761)** | | |
|  | Laborers (laborers, n.e.c. ^1^) | 39.9% |
|  | Tailors, dress makers, sewers, upholsterers & related workers | 30.0% |
|  | Tobacco preparers & tobacco product makers | 10.0% |
|  | Spinners, weavers, knitters, dyers and related workers | 4.6% |
|  | Food and beverage processors | 3.1% |
|  | Stone cutters & carvers | 2.7% |
|  | Jewelry & precious metal workers and metal engravers | 1.3% |
|  | Tanners, fellmongers and pelt dressers | 0.9% |
|  | Miners, quarrymen, well drillers & related workers | 0.9% |
|  | Chemical processors and related workers | 0.9% |
|  | Wood preparation workers and paper makers | 0.8% |
|  | Machinery fitters, machine assemblers and precession instrument makers | 0.8% |
|  | Carpenters, cabinet & related wood workers | 0.6% |
|  | Shoemakers & leather goods makers | 0.5% |
|  | Paper & paper board products makers | 0.4% |
|  | Rubber and plastic product makers workers | 0.4% |
|  | Metal processors | 0.4% |
|  | Glass formers, potters & related workers | 0.4% |
|  | Electrical fitters & related electrical & electronic workers | 0.4% |
|  | Transport equipment operators (driver) | 0.3% |
|  | Blacksmiths, tool makers and machine tools operators | 0.2% |
|  | Painters | 0.2% |
|  | Plumbers, welders, sheet metal & structural metal preparers and erectors | 0.2% |
|  | Stationery engines and related equipment operators, oilers | 0.3% |

^1^Not elsewhere classified
