# Supplementary material for: Planning for work: Exploring the relationship between contraceptive use and women’s sector-specific employment in India
Source: PLoS One. 2021 Mar 11;16(3):e0248391. doi: 10.1371/journal.pone.0248391 (PMC7951869; doi:10.1371/journal.pone.0248391)
Supplement: S2 Table — (DOCX) [file pone.0248391.s002.docx]

**S2 Table. Descriptive summary of variables across current employment sector among 18-49 year old women in India, 2015-16.**

|  |  | Current employment sector | | | | | |
| --- | --- | --- | --- | --- | --- | --- | --- |
|  | Total^1^ (n=48,232) | No current employment (n=37,600) | Professional sector (n=1,232) | Clerical or sales sectors (n=770) | Agricultural sector (n=4,641) | Services sector (n=1,228) | Production sector(n=2,761) |
|  | % (95% CI) | % (95% CI) | % (95% CI) | % (95% CI) | % (95% CI) | % (95% CI) | % (95% CI) |
| Total |  | 76.0 (75.3-76.7) | 2.6 (2.4-2.9) | 1.6 (1.5-1.8) | 10.5 (10.1-11.0) | 2.8 (2.6-3.1) | 6.4 (6.0-6.9) |
| **Current contraceptive use** | | | | | | | |
| None | 23.7 (23.1 - 24.2) | 84.1 (83.1 - 85.1) | 3.0 (2.5 - 3.5) | 1.3 (1.0 - 1.7) | 5.6 (5.1 - 6.1) | 1.9 (1.5 - 2.3) | 4.1 (3.5 - 4.7) |
| Female sterilization | 51.9 (51.2 - 52.7) | 69.0 (68.0 - 70.0) | 1.9 (1.6 - 2.1) | 1.9 (1.6 - 2.1) | 16.1 (15.3 - 16.9) | 3.5 (3.1 - 3.9) | 7.7 (7.1 - 8.3) |
| IUD | 2.1 (1.9 - 2.2) | 78.6 (74.5 - 82.7) | 7.2 (4.6 - 9.8) | 1.2 (0.5 - 2.0) | 3.6 (2.3 - 4.9) | 3.4 (2.0 - 4.8) | 6.0 (2.7 - 9.4) |
| Condom | 8.2 (7.7 - 8.6) | 84.8 (83.0 - 86.6) | 5.7 (4.2 - 7.3) | 1.1 (0.7 - 1.5) | 2.2 (1.7 - 2.7) | 1.8 (1.1 - 2.5) | 4.4 (3.5 - 5.2) |
| Pill | 5.8 (5.5 - 6.2) | 83.6 (81.0 - 86.1) | 2.0 (1.2 - 2.8) | 1.9 (1.0 - 2.8) | 3.4 (2.4 - 4.4) | 1.9 (1.1 - 2.7) | 7.2 (5.0 - 9.5) |
| Rhythm | 4.8 (4.5 - 5.2) | 81.4 (78.9 - 84.0) | 2.3 (1.6 - 3.0) | 1.5 (0.7 - 2.2) | 5.4 (4.2 - 6.7) | 2.9 (1.3 - 4.4) | 6.4 (4.7 - 8.2) |
| Withdrawal | 3.5 (3.2 - 3.8) | 83.2 (80.5 - 86.0) | 3.4 (2.0 - 4.8) | 1.3 (0.6 - 2.0) | 3.0 (2.1 - 4.0) | 2.1 (1.3 - 2.9) | 6.9 (4.8 - 9.0) |
| **Reproductive history** | | | | | | | |
| Parity |  |  |  |  |  |  |  |
| No births | 7.2 (6.8 - 7.5) | 87.4 (85.7 - 89.1) | 4.0 (3.0 - 5.1) | 0.8 (0.4 - 1.2) | 3.3 (2.6 - 4.1) | 1.3 (0.7 - 1.9) | 3.2 (2.3 - 4.1) |
| 1 birth | 15.6 (15.0 - 16.3) | 85.1 (83.6 - 86.6) | 4.3 (3.4 - 5.3) | 1.6 (1.1 - 2.1) | 3.6 (3.0 - 4.1) | 1.8 (1.3 - 2.3) | 3.6 (2.8 - 4.4) |
| 2 births | 37.3 (36.6 - 38.0) | 74.7 (73.7 - 75.8) | 3.2 (2.8 - 3.6) | 1.9 (1.5 - 2.2) | 10.3 (9.6 - 11.1) | 3.2 (2.8 - 3.6) | 6.7 (6.0 - 7.4) |
| 3 or more births | 39.9 (39.2 - 40.6) | 71.6 (70.6 - 72.6) | 1.2 (1.0 - 1.4) | 1.6 (1.3 - 1.8) | 14.7 (14.0 - 15.4) | 3.1 (2.8 - 3.5) | 7.8 (7.2 - 8.5) |
| Living sons |  |  |  |  |  |  |  |
| None | 22.3 (21.7 - 22.9) | 81.6 (80.4 - 82.7) | 3.8 (3.3 - 4.4) | 1.4 (1.1 - 1.8) | 6.5 (5.8 - 7.2) | 1.9 (1.5 - 2.2) | 4.9 (4.1 - 5.6) |
| Any | 77.7 (77.1 - 78.3) | 74.4 (73.6 - 75.2) | 2.3 (2.0 - 2.6) | 1.7 (1.5 - 1.9) | 11.7 (11.1 - 12.2) | 3.1 (2.8 - 3.4) | 6.9 (6.3 - 7.4) |
| **Sociodemographics** | | | | | | | |
| Age (mean years) | 33.5 (33.4 - 33.6) | 33.0 (32.8 - 33.1) | 34.8 (34.1 - 35.4) | 35.9 (35.0 - 36.8) | 35.7 (35.4 - 35.9) | 36.1 (35.5 - 36.7) | 34.6 (34.2 - 35.0) |
| Education (mean years) | 6.5 (6.4 - 6.6) | 6.9 (6.8 - 7.0) | 13.7 (13.3 - 14.1) | 7.8 (7.2 - 8.5) | 2.9 (2.7 - 3.1) | 6.4 (5.9 - 6.8) | 4.9 (4.6 - 5.2) |
| Number of household residents (mean) | 5.5 (5.5 - 5.6) | 5.6 (5.6 - 5.7) | 5.1 (4.9 - 5.4) | 5.0 (4.7 - 5.2) | 5.3 (5.2 - 5.3) | 5.0 (4.8 - 5.2) | 5.1 (5.0 - 5.2) |
| Household dependency percentage (mean) | 34.5 (34.2 - 34.8) | 34.7 (34.3 - 35.0) | 33.4 (31.6 - 35.1) | 30.4 (27.9 - 32.9) | 34.5 (33.7 - 35.3) | 30.2 (28.3 - 32.0) | 35.6 (34.2 - 36.9) |
| Residence |  |  |  |  |  |  |  |
| Rural | 61.9 (61.1 - 62.6) | 73.8 (73.0 - 74.7) | 1.6 (1.4 - 1.8) | 1.2 (1.0 - 1.4) | 15.6 (14.9 - 16.3) | 2.1 (1.8 - 2.3) | 5.7 (5.2 - 6.1) |
| Urban | 38.1 (37.4 - 38.9) | 79.5 (78.3 - 80.7) | 4.3 (3.7 - 4.8) | 2.3 (1.9 - 2.7) | 2.2 (1.8 - 2.6) | 4.0 (3.5 - 4.6) | 7.7 (6.7 - 8.6) |
| SC/ST or OBC |  |  |  |  |  |  |  |
| Not SC/ST or OBC | 29.7 (28.8 - 30.6) | 82.4 (81.2 - 83.7) | 3.6 (3.0 - 4.2) | 1.7 (1.4 - 2.1) | 4.8 (4.1 - 5.4) | 2.5 (1.9 - 3.0) | 4.9 (4.1 - 5.7) |
| SC/ST | 27.0 (26.1 - 27.9) | 67.3 (66.0 - 68.6) | 2.0 (1.6 - 2.3) | 1.4 (1.1 - 1.7) | 17.1 (16.0 - 18.2) | 3.6 (3.1 - 4.0) | 8.7 (7.8 - 9.6) |
| OBC | 43.3 (42.4 - 44.1) | 77.0 (76.1 - 77.9) | 2.4 (2.1 - 2.7) | 1.7 (1.4 - 2.0) | 10.3 (9.7 - 11.0) | 2.6 (2.2 - 2.9) | 6.0 (5.5 - 6.5) |
| Wealth quintile |  |  |  |  |  |  |  |
| Poorest | 13.4 (12.9 - 13.8) | 69.9 (68.3 - 71.5) | 0.3 (0.1 - 0.4) | 0.7 (0.5 - 0.9) | 20.2 (18.8 - 21.6) | 1.5 (1.1 - 2.0) | 7.4 (6.5 - 8.3) |
| Poorer | 17.7 (17.2 - 18.3) | 70.8 (69.3 - 72.3) | 0.8 (0.5 - 1.1) | 0.9 (0.6 - 1.2) | 17.8 (16.6 - 19.0) | 2.5 (2.1 - 3.0) | 7.2 (6.1 - 8.3) |
| Middle | 20.5 (19.9 - 21.1) | 72.4 (71.0 - 73.8) | 1.3 (1.0 - 1.5) | 1.5 (1.1 - 1.8) | 14.4 (13.3 - 15.6) | 3.6 (3.0 - 4.2) | 6.8 (5.9 - 7.7) |
| Richer | 23.1 (22.4 - 23.9) | 78.3 (77.1 - 79.6) | 3.0 (2.4 - 3.6) | 2.1 (1.7 - 2.6) | 6.0 (5.3 - 6.7) | 3.6 (3.1 - 4.2) | 6.9 (6.1 - 7.7) |
| Richest | 25.3 (24.4 - 26.1) | 83.7 (82.4 - 85.0) | 6.0 (5.3 - 6.7) | 2.3 (1.8 - 2.7) | 1.2 (0.9 - 1.6) | 2.3 (1.8 - 2.7) | 4.6 (3.6 - 5.6) |
| **Gender equity** | | | | | | | |
| Age at first marriage or cohabitation |  |  |  |  |  |  |  |
| <15 | 15.2 (14.7 - 15.7) | 67.5 (65.9 - 69.2) | 0.9 (0.6 - 1.2) | 1.7 (1.2 - 2.2) | 17.2 (15.9 - 18.5) | 4.0 (3.1 - 4.8) | 8.8 (7.4 - 10.1) |
| 15-17 | 29.1 (28.5 - 29.8) | 75.3 (74.1 - 76.4) | 0.9 (0.7 - 1.1) | 1.4 (1.1 - 1.8) | 12.7 (11.8 - 13.5) | 2.5 (2.1 - 2.9) | 7.2 (6.4 - 8.0) |
| 18+ | 55.7 (55.0 - 56.5) | 78.7 (77.9 - 79.5) | 4.1 (3.6 - 4.5) | 1.7 (1.5 - 1.9) | 7.5 (7.1 - 8.0) | 2.7 (2.3 - 3.0) | 5.4 (4.9 - 5.9) |
| Marital education gap |  |  |  |  |  |  |  |
| Equally educated | 28.3 (27.7 - 28.9) | 71.9 (70.7 - 73.2) | 2.7 (2.3 - 3.1) | 1.5 (1.2 - 1.9) | 13.5 (12.6 - 14.3) | 2.9 (2.4 - 3.4) | 7.5 (6.6 - 8.4) |
| Wife is more educated | 23.6 (23.0 - 24.2) | 76.1 (74.8 - 77.4) | 5.0 (4.3 - 5.7) | 2.3 (1.9 - 2.8) | 7.0 (6.3 - 7.8) | 3.6 (3.0 - 4.1) | 5.9 (5.2 - 6.7) |
| Husband is more educated | 48.1 (47.4 - 48.8) | 78.4 (77.5 - 79.2) | 1.5 (1.2 - 1.7) | 1.3 (1.1 - 1.5) | 10.5 (9.9 - 11.1) | 2.4 (2.1 - 2.7) | 6.0 (5.4 - 6.6) |
| Freedom of movement |  |  |  |  |  |  |  |
| Restricted access/  accompaniment required to 1+ locations | 54.4 (53.5 - 55.2) | 81.2 (80.5 - 82.0) | 1.6 (1.3 - 1.9) | 1.0 (0.9 - 1.2) | 9.4 (8.8 - 9.9) | 1.9 (1.6 - 2.2) | 4.9 (4.5 - 5.4) |
| Freedom of movement to all three locations | 45.6 (44.8 - 46.5) | 69.8 (68.7 - 70.9) | 3.9 (3.5 - 4.3) | 2.3 (2.0 - 2.6) | 11.9 (11.2 - 12.6) | 3.9 (3.5 - 4.4) | 8.2 (7.4 - 9.0) |
| Decision-making involvement |  |  |  |  |  |  |  |
| Not involved in 1+ decisions | 34.8 (34.0 - 35.6) | 80.4 (79.5 - 81.4) | 1.5 (1.2 - 1.8) | 1.1 (0.9 - 1.4) | 10.0 (9.3 - 10.7) | 2.0 (1.6 - 2.3) | 4.9 (4.4 - 5.5) |
| Involved in all three decisions | 65.2 (64.4 - 66.0) | 73.7 (72.8 - 74.5) | 3.2 (2.9 - 3.6) | 1.9 (1.6 - 2.1) | 10.8 (10.2 - 11.3) | 3.3 (2.9 - 3.6) | 7.2 (6.6 - 7.8) |

^1^ Column percentages. All other percentages are row.
