# Supplementary material for: Planning for work: Exploring the relationship between contraceptive use and women’s sector-specific employment in India
Source: PLoS One. 2021 Mar 11;16(3):e0248391. doi: 10.1371/journal.pone.0248391 (PMC7951869; doi:10.1371/journal.pone.0248391)
Supplement: S3 Table — (DOCX) [file pone.0248391.s003.docx]

**S3 Table. Multivariable, multinomial regression assessing the associations between current contraceptive use and current employment sector among married women aged 18-49 in India, 2015-16.**

|  | Professional sector (vs. no current employment) | | Clerical or sales sectors (vs. no current employment) | | Agricultural sector (vs. no current employment) | | Services sector (vs. no current employment) | | Production sector (vs. no current employment) | |
| --- | --- | --- | --- | --- | --- | --- | --- | --- | --- | --- |
|  | aRRR  (95% CI) | p-value | aRRR  (95% CI) | p-value | aRRR  (95% CI) | p-value | aRRR  (95% CI) | p-value | aRRR  (95% CI) | p-value |
| Current contraception |  |  |  |  |  |  |  |  |  |  |
| None | REF |  | REF |  | REF |  | REF |  | REF |  |
| Female sterilization | 0.78 (0.58 - 1.05) | 0.10 | 0.97 (0.70 - 1.35) | 0.86 | 1.51 (1.31 - 1.74) | <0.001 | 1.11 (0.83 - 1.49) | 0.49 | 1.46 (1.19 - 1.78) | <0.001 |
| IUD | 1.89 (1.21 - 2.93) | 0.01 | 0.78 (0.38 - 1.61) | 0.51 | 1.36 (0.91 - 2.03) | 0.14 | 1.46 (0.90 - 2.37) | 0.12 | 1.53 (0.82 - 2.86) | 0.18 |
| Condom | 1.37 (0.86 - 2.20) | 0.18 | 0.75 (0.46 - 1.24) | 0.26 | 0.87 (0.66 - 1.14) | 0.31 | 0.86 (0.54 - 1.38) | 0.54 | 1.15 (0.87 - 1.51) | 0.32 |
| Pill | 1.12 (0.68 - 1.87) | 0.65 | 1.53 (0.87 - 2.70) | 0.14 | 0.89 (0.62 - 1.28) | 0.55 | 0.77 (0.44 - 1.35) | 0.36 | 1.41 (1.00 - 1.99) | 0.05 |
| Rhythm | 0.90 (0.58 - 1.41) | 0.64 | 1.09 (0.61 - 1.95) | 0.76 | 1.45 (1.08 - 1.96) | 0.01 | 1.23 (0.75 - 2.00) | 0.41 | 1.40 (0.99 - 1.97) | 0.06 |
| Withdrawal | 1.13 (0.70 - 1.83) | 0.62 | 0.88 (0.48 - 1.60) | 0.67 | 0.96 (0.67 - 1.38) | 0.83 | 0.87 (0.53 - 1.42) | 0.57 | 1.54 (1.08 - 2.21) | 0.02 |
| **Reproductive history** | | | | | | | | | | |
| Parity |  |  |  |  |  |  |  |  |  |  |
| No births | REF |  | REF |  | REF |  | REF |  | REF |  |
| 1 birth | 0.57 (0.38 - 0.85) | 0.01 | 1.23 (0.63 - 2.40) | 0.55 | 0.87 (0.65 - 1.17) | 0.35 | 0.74 (0.41 - 1.32) | 0.30 | 0.71 (0.48 - 1.04) | 0.08 |
| 2 births | 0.60 (0.41 - 0.88) | 0.01 | 1.16 (0.61 - 2.20) | 0.65 | 1.13 (0.85 - 1.49) | 0.40 | 0.91 (0.53 - 1.56) | 0.73 | 0.98 (0.70 - 1.39) | 0.93 |
| 3+ births | 0.69 (0.45 - 1.06) | 0.09 | 1.23 (0.60 - 2.50) | 0.58 | 1.07 (0.81 - 1.42) | 0.63 | 0.88 (0.50 - 1.57) | 0.68 | 0.97 (0.67 - 1.41) | 0.88 |
| **Sociodemographics** | | | | | | | | | | |
| Age (years) | 1.44 (1.27 - 1.63) | <0.001 | 1.27 (1.09 - 1.48) | 0.002 | 1.26 (1.18 - 1.33) | <0.001 | 1.49 (1.33 - 1.66) | <0.001 | 1.28 (1.19 - 1.39) | <0.001 |
| Education (mean years) | 1.44 (1.37 - 1.52) | <0.001 | 1.04 (1.01 - 1.08) | 0.02 | 0.91 (0.89 - 0.92) | <0.001 | 1.03 (1.00 - 1.05) | 0.03 | 0.95 (0.93 - 0.96) | <0.001 |
| Residence |  |  |  |  |  |  |  |  |  |  |
| Rural | REF |  | REF |  | REF |  | REF |  | REF |  |
| Urban | 0.97 (0.78 - 1.21) | 0.80 | 1.31 (1.01 - 1.70) | 0.04 | 0.22 (0.18 - 0.27) | <0.001 | 2.11 (1.66 - 2.68) | <0.001 | 1.75 (1.46 - 2.10) | <0.001 |
| Wealth quintile |  |  |  |  |  |  |  |  |  |  |
| Poorest | 1.38 (0.78 - 2.42) | 0.27 | 0.90 (0.54 - 1.52) | 0.70 | 13.69 (9.53 - 19.68) | <0.001 | 2.85 (1.86 - 4.38) | <0.001 | 2.78 (2.04 - 3.77) | <0.001 |
| Poorer | 1.74 (1.15 - 2.65) | 0.01 | 0.91 (0.60 - 1.40) | 0.68 | 8.06 (5.69 - 11.41) | <0.001 | 3.29 (2.30 - 4.70) | <0.001 | 2.30 (1.71 - 3.08) | <0.001 |
| Middle | 1.40 (1.05 - 1.87) | 0.02 | 1.15 (0.81 - 1.64) | 0.44 | 5.20 (3.71 - 7.28) | <0.001 | 3.48 (2.53 - 4.80) | <0.001 | 1.94 (1.49 - 2.54) | <0.001 |
| Richer | 1.60 (1.23 - 2.09) | <0.001 | 1.19 (0.88 - 1.61) | 0.26 | 2.33 (1.65 - 3.28) | <0.001 | 2.29 (1.75 - 2.99) | <0.001 | 1.56 (1.22 - 2.00) | <0.001 |
| Richest | REF |  | REF |  | REF |  | REF |  | REF |  |
| **Gender equity** |  |  |  |  |  |  |  |  |  |  |
| Age at first marriage or cohabitation |  |  |  |  |  |  |  |  |  |  |
| <15 | 1.39 (0.88 - 2.18) | 0.16 | 1.46 (1.03 - 2.06) | 0.04 | 1.28 (1.12 - 1.46) | <0.001 | 1.73 (1.32 - 2.25) | <0.001 | 1.32 (1.07 - 1.63) | 0.01 |
| 15-17 | 0.81 (0.60 - 1.10) | 0.18 | 1.10 (0.82 - 1.47) | 0.51 | 1.14 (1.03 - 1.27) | 0.01 | 1.05 (0.84 - 1.31) | 0.66 | 1.13 (0.98 - 1.31) | 0.09 |
| 18+ | REF |  | REF |  | REF |  | REF |  | REF |  |

aRRR = adjusted relative risk ratio. Results adjust for age squared and a state/union territory fixed effect, as well as all variables shown.
